# "I'm not a volunteer; I'm an equal player": the social factor of quality of life in mixed ability basketball players

**DOI:** 10.3389/fspor.2026.1770991

**Published:** 2026-04-13

**Authors:** Pablo Elipe-Lorenzo, Rui Resende, Pelayo Diez-Fernández, Óscar Gonzalo, Brais Ruibal-Lista, Aitor Coca, Carla da-Silva, Sergio López-García

**Affiliations:** 1 Faculty of Education, Universidad Pontificia de Salamanca, Salamanca, Spain; 2 Research Group in Physical Activity, Sport and Health (GIADES), Faculty of Education, Universidad Pontificia de Salamanca, Salamanca, Spain; 3 Escola Superior de Desporto e Lazer do Instituto Politécnico de Viana do Castelo, Viana do Castelo, Portugal; 4 Sport Physical Activity and Health Research & Innovation Center (SPRINT), Viana do Castelo, Portugal; 5 Department of Functional Biology, Universidad de Oviedo, Oviedo, Spain; 6 EUM Fray Luis de León, Universidad Católica de Ávila, Valladolid, Spain; 7 Department of Physical Activity and Sport Sciences, Faculty of Health Sciences, Universidad Euneiz, Vitoria-Gasteiz, Spain; 8 International Mixed Ability Sports (IMAS Spain), Vitoria-Gasteiz, Spain; 9 Research Group in Sport Sciences (INCIDE), Universidade da Coruña, A Coruña, Spain

**Keywords:** communities, grassroots sport, inclusive sport, mixed abilities, shared citizenship, disability, quality of life

## Abstract

**Introduction:**

Despite regulatory and conceptual progress in the field of inclusion, people with disabilities continue to face significant barriers which limit their full participation in mainstream sports and affect key social factors of quality of life. Within this context, the Mixed Ability (MA) model represents an alternative where people with disabilities can be included into grassroots sports settings without the need to modify rules or implement classification or identification systems. This study aimed to analyse perceived changes in the social factor of MA basketball players, as well as to identify current needs and challenges related to their participation.

**Methods:**

A qualitative methodology was employed, using individual semi-structured interviews with a convenience sample. Eleven players from a basketball MA club took part, distributed across two men's teams and one women's team.

**Results:**

Three categories were identified: “interpersonal relationships”, “social inclusion and rights”, and “barriers, future, and proposed changes”. Participation in MA basketball teams has fostered social connection, greater group cohesion, a natural support network, a sense of belonging, and a perception of equality between people with and without disabilities. However, relevant obstacles were also identified, such as the short duration and infrequent occurrence of training sessions and competitions, the need to rejuvenate the team, and issues related to communication and lack of awareness.

**Discussion:**

The findings suggest that MA may contribute to the development of interpersonal relationships, social inclusion, and the recognition of the rights of people with disabilities. At the same time, they point to the importance of addressing structural, social, and organizational conditions in order to support more inclusive and sustainable sporting environments. In this sense, inclusion emerges not as a fixed outcome, but as a dynamic and ongoing process shaped by contextual factors and the quality of relationships within sport.

## 1 Introduction

Building inclusive communities has become a priority objective for numerous institutions and international bodies. The United Nations (UN) 2030 Agenda emphasises the importance of reducing inequalities as part of the Sustainable Development Goals (SDGs), while the Convention on the Rights of Persons with Disabilities (CRPD) (1) highlights their full and effective participation in society as a core principle. Social inclusion of people with disabilities is defined as active participation in community life through meaningful social relationships, particularly with people without disabilities and non-professional individuals nor family members, which contribute to the development of socially integrated support networks (2, 3). This participation involves genuine access to community activities, social roles, and everyday relationships in mainstream settings, as well as the use of community resources and facilities, promoting social interaction beyond segregated environments (2, 4). From this perspective, social inclusion also entails recognition of the individual as a competent and valued member of the community, fostering a sense of belonging, with opportunities to assume socially recognized roles, participate in social and economic life, and receive appropriate natural supports that enable reciprocal and meaningful relationships (5–8). Furthermore, the context plays a key role, as the development of links between natural supports, people with disabilities, and the wider community facilitates processes of belonging, community living, and access to activities and opportunities (9, 10). Social inclusion is considered a key dimension of quality of life (QoL), as being part of society strongly influences one's sense of well-being (11). The CRPD recognises social inclusion as a fundamental right, linked to the opportunity to actively engage in global activities and decision-making processes (11). In this sense, participation in community is an essential dimension of human functioning (12, 13) and a central component of QoL for people with disabilities (14).

The social factor within the QoL paradigm includes three main dimensions: interpersonal relationships (IR), rights (RI), and social inclusion (SI) (15). IR refer to the presence, quality, and stability of social networks, including friendships and intimate relationships (16, 17). RI encompass formal legal protections and the everyday experience of being treated as a rights-holder in decisions affecting one's life, including participation in community (1, 14, 18). SI has been addressed above. These three dimensions are operationalized through eight factors that constitute the overall QoL construct: emotional well-being, interpersonal relationships, material well-being, personal development, physical well-being, self-determination, social inclusion, and rights (15, 19).

The concept of QoL is understood as a universal construct, shaped by the same underlying dimensions and relationships across all people. QoL emerges when people's needs are adequately addressed and when they have opportunities to engage in meaningful life activities that promote personal fulfillment. It encompasses both subjective perceptions and objective living conditions (6, 15). Furthermore, QoL is conceived as a multidimensional phenomenon, resulting from the dynamic interaction between personal and environmental influences. Needs are understood as discrepancies between a person's current situation and desired outcomes in each QoL domain. In this sense, social inclusion, independence, and well-being are interrelated factors within the QoL paradigm, highlighting the importance of personal and environmental supports to achieve a fulfilled life (6).

In the Spanish national context, where this study is based, legislation such as (20), which regulates the rights of persons with disabilities and their social inclusion, and Law 39/2022 (21) on Sport, reinforces the promotion of social cohesion and equality in living conditions. Notably, the last one highlights inclusive sport as a practice that promotes joint activities between people with and without disabilities on equal terms. Nevertheless, in Europe, and particularly in Spain, the development of community services remains limited (22–24). In this scenario, sport emerges as a powerful tool for promoting SI. Recognised by the CRPD as a fundamental right (1), sport has the potential to reduce the barriers faced by people with disabilities and to foster their active participation. Indeed, the CRPD explicitly recognises the right to participate in mainstream sporting activities. Like anyone else, they also wish to participate in inclusive clubs rather than in separate structures (25). However, rights alone are insufficient unless individuals are provided with genuine opportunities to put them into practice (15). Despite regulatory and conceptual progress, people with disabilities still face significant barriers that limit their full inclusion into the community, contributing to higher rates of physical inactivity (26, 27). In many cases, they remain in a “separate social space”, restricted to interactions with family, cohabitants, or support professionals, which limits their active participation as citizens (28). Such dynamics perpetuate exclusion and violate fundamental rights, resulting in “flat, unwanted, unfulfilled lives” (29 p. 2). In sports context, genuine inclusion involves enabling people with disabilities to take part in sport alongside people without disabilities under shared, standardised rules, requiring only minimal and necessary adjustments, and allowing individuals to exercise choice to choose how and with whom to participate (30, 31). Kiuppis (32) highlights that the concept of inclusion in sport is often used superficially, noting that many policies invoke it rhetorically without examining the actual forms of participation being provided. Clubs and organisations frequently apply the term inclusion to initiatives that are in fact integrative or segregated, creating considerable scope for labelling almost any programme as “inclusive” (33). Also, the sporting context may reproduce power hierarchies and maintain restrictive views on disability (34). Therefore, the sports domain remains segregated, underlining the need for more open, accessible, and inclusive environments (35). In this regard, the right to be included in mainstream sport with the necessary adjustments to enable fair and meaningful participation, is fundamental (36). Sports clubs, in this sense, represents a potential space for strengthening social cohesion and promoting community support networks (37). However, to move towards inclusion, it is essential to ensure that people with disabilities not only take part in these spaces but also have an active and meaningful role in activities (36, 38).

In this context, the Mixed Ability (MA) model, developed and promoted by the international non-profit organisation International Mixed Ability Sports (IMAS), has been described as one approach to inclusion in sport. With an international presence and registration in Spain, IMAS supports the joint participation of people without or with any type of disabilities within mainstream sports clubs (39). The model is characterised by shared participation under standard sporting regulations, with the use of reasonable adjustments when necessary, and without the introduction of formal identification systems based on disability (40). In this respect, it differs from other segregated models or approaches that include people without disabilities but restrict participation to a single type of disability while excluding others, as is the case with Unified Sports promoted by Special Olympics. In this model, participation remains highly structured, with team composition based on ability level, and participants categorised as either “athletes” (with disabilities) or “partners” (without disabilities) (41). While such initiatives can enhance visibility and empowerment for some athletes, they may also contribute to the perception that people with disabilities are subject to classification systems that influence their opportunities to participate alongside others (98–100). The MA model, by contrast, offers an alternative approach in which players with and without disabilities compete and participate together under shared rules and roles, without relying on classification (40).

In the Spanish context, empirical studies conducted by Elipe-Lorenzo et al. (42) and da-Silva (43) have begun to provide initial evidence regarding the implementation of MA rugby and its effects on participants. Similarly, research in the United Kingdom, Italy and Argentina has reported data on the development of the model within community-based settings (39, 44–46). However, further research is required to advance understanding of how MA operates in sporting contexts beyond rugby. Given that the current evidence base is largely derived from rugby settings, there remains a limited understanding of participants’ lived experiences and perceptions of social aspects in other sports within the model. Accordingly, in response to the limited availability of mainstream sporting opportunities for people with disabilities, and to the need for a deeper understanding of lived experiences within inclusive sporting contexts, this study posed the following research question: How do players perceive their social inclusion, rights, and interpersonal relationships as a result of participating in MA basketball? Therefore, this study aimed to explore perceived changes as well as the current needs and challenges related to these social factors within the QoL paradigm among MA basketball players.

## 2 Method

This qualitative research was conducted through semi-structured interviews. This data collection method allows for an in-depth exploration of participants’ experiences and their context, understood as the individual and subjective representation of how people perceive and interpret their world (47, 48).

### 2.1 Procedure

The research procedure design considered participants’ relationship with the topic in question. The following precautions were taken into account (49): (a) Access to group members, (b) The profile of the issue, (c) The perspectives and discursive capacities of the group members, (d) Their identification with the research topic, (e) Ethical considerations in data collection, processing and usage, (f) The possibility of recording the sessions, (g) Representation of the research process experience and group members’ accounts, (h) The analysis process, and (i) The production of a written report.

The interviews were conducted by two researchers familiar with the concept of the social factor within the QoL paradigm and the MA sports model. They were carried out during October and November 2024, with an average duration of 61 min per interview, ranging from 40 min to 80 min. Interviews took place at the University of Vitoria-Gasteiz (EUNEIZ), chosen for its suitable facilities and proximity to Saski Baskonia's stadium, which facilitated participant comfort and accessibility. All interviews were audio recorded using a Marantz Professional PMD661 MKIII to ensure accuracy in transcription and later analysis. The audio files were transcribed verbatim into a 405-page Word document (Cambria, font size 11, line spacing 1.15). All identifiable information was then removed to ensure participant anonymity.

During data collection, the importance of emotional relationships was highlighted as a means of gaining field access (50). Building a bond of trust was prioritised to enable effective communication during the interviews (51–53). In this regard, both researchers actively engaged with the team for one month, participating in training sessions, matches, and social gatherings. This allowed for a deep understanding of the team's reality and helped establish a strong bond of trust between players and researchers. This facilitated the inclusion and understanding of the participants’ specific needs (54). Furthermore, as noted in the literature, this immersion enabled researchers to identify and adjust necessary adaptations for the interviews (55).

At the beginning of the first training session, participants were invited to indicate their interest in the study via a Google Forms® questionnaire, through which 11 participants wanted to participate individually. Those meeting the inclusion criteria were informed of the study's aims and the data collection procedures. Interviews were conducted until data saturation was achieved in the club setting—when responses became repetitive and no new relevant ideas emerged for analysis (56, 57).

### 2.2 Participants

A total of 11 players from three MA Baskonia basketball teams (two men's and one women's team) took part in the study. The sample consisted of four women and seven men, all of whom chose to participate individually. Of these, eight participants had a disability (including down syndrome, deafness combined with autism, and acquired brain injury), while three participants did not report any disability. Participants’ ages ranged from 20 to 55 years, with a mean age of 34.6. Six of them had four years of experience in MA basketball, while the remaining five had three years. Only two participants requested to be accompanied by a support person during the interview (Table 1).

**Table 1:** Participant data from individual interviews.

Participants	Age	Type of disability	Years playing MA	Team	Type of support
P1	20	Down syndrome	4	Male	
P2	55	No disability	3	Female	
P3	48	No disability	4	Male	
P4	48	No disability	3	Male	
P5	30	Down syndrome	4	Female	Support person
P6	35	Down syndrome	4	Male	
P7	39	Down syndrome	4	Male	
P8	26	Down syndrome	3	Female	
P9	28	Deafness and autism	3	Male	Sign language interpreter
P10	32	Acquired brain injury	3	Male	
P11	20	Down syndrome	4	Female	

Own elaboration.

Participants were selected through convenience sampling. Inclusion criteria focused on players aged 18 or over, with or without disabilities, who had been practising MA basketball for at least one year. This decision aligns with the philosophical and operational principles of the MA model, which rejects segregation and promotes joint participation without classification or differential adaptations. Including both people with and without disabilities in the qualitative study reflects the model's commitment to exploring relational dynamics, mutual support, and shared sporting experiences in an inclusive and horizontal environment. The initial contact was made with Saski Baskonia, which acted as a key link between the researchers and the players, consistently facilitating communication and interaction throughout the process.

To ensure inclusivity, a variety of strategies and flexible methods were employed, taking into account individual participants’ needs and including the use of accessible language (58). All players were invited to participate through a Google Forms® questionnaire, which gathered sociodemographic information such as name, age, type of disability, and years of experience in MA basketball. It also allowed players to indicate their participation preferences (individual or group) and required support. These measures aimed to foster voluntary participation and ensure that no one felt obligated to take part in the study (59). These inclusive strategies supported accessible communication and helped clarify ambiguous responses, ensuring active and meaningful participation from all involved (60).

### 2.3 Design and materials

The method used in this study consisted of Individual Interviews (II), aimed at exploring a specific set of questions (61) related to MA basketball. A structured interview guide was developed to assist the moderator in directing the data collection process. This technique enabled researchers to listen to participants’ socially constructed viewpoints and beliefs (62), while allowing participants relative freedom to discuss the topics raised.

The interview guide was carefully developed by the research team based on the study's objectives and the QoL paradigm (15), ensuring alignment between the questions and the social factor domains central to the research. The guide intentionally avoided direct questions framed around “needs” or “challenges” in order not to impose a deficit-oriented perspective on participants. Instead, each question invited them to narrate their experiences in relation to the social factor domains (IR, SI and RI), allowing them to introduce facilitators, barriers, concerns and aspirations in their own terms.

Although no previous study had employed the exact same questions, their formulation drew on existing literature on MA sport settings by Corazza and Dyer (39), da-Silva (43) and Dyer and Sandford (46) and on the QoL paradigm by Schalock et al. (19) and Verdugo et al. (15), particularly on the social factor and its three domains, which included exemplary indicators such as social networks, friendships, community involvement, support systems and human and legal rights. The interview guide underwent a rigorous validation process by a committee of seven professionals from various fields. This included a qualitative methodology expert from the Instituto Politécnico de Viana do Castelo, three MA specialists from IMAS, and three experts in QoL, inclusive environments, and disability from the Instituto Universitario de Integración en la Comunidad (INICO). Among the members was one of the pioneers of the QoL paradigm for people with intellectual and/or developmental disabilities. Subsequently, the guide was adapted into Easy Read format by two INICO experts and validated by 15 individuals with intellectual disabilities. This process ensured the interviews were comprehensible, accessible, and aligned with the study's aims, enabling a clear and meaningful exploration of the subject matter. Each question was, therefore, grounded in both theoretical and applied knowledge to ensure relevance to the research aims.

The final semi-structured interview guide consisted of 11 questions, used to structure the conversation (Table 2). This was key in maintaining organisation and thematic coherence related to QoL (47). The questions corresponded to the social factor from the QoL paradigm (15). Participants were also encouraged to talk about their experiences with MA basketball, as well as their perceptions of social relationships, fears, moods, and other relevant aspects related to the needs and challenges associated with their involvement in the sports environment.

**Table 2:** Semi-structured interview guide addressing the social factor within the quality of life paradigm.

In relation to the Social Factor…	Dimension
**1. What does Mixed Ability basketball mean to you?**	SI; RI; IR
**2. Why do you play Mixed Ability basketball?**	SI; RI; IR
**3. How do your teammates treat you?**	IR; RI
**4. Do you receive any support during the game?**	SI; IR
**5. What changes in your daily life have come from being part of this Mixed Ability basketball team?**	SI; RI; IR
**6. When you are not playing basketball, do you spend time with your teammates?**	IR; SI
**7. Would you like to do more activities with your teammates outside of basketball?**	IR; SI
**8. Why do you think it is important to follow the official rules of basketball?**	RI; SI
**9. What are the best and worst things about playing basketball with Baskonia?**	SI; RI
**10. How do you see the team in the future?**	SI; RI
**11. What would you change about the Mixed Ability team?**	RI; SI; IR

Own elaboration. SI, Social Inclusion; RI, Rights; IR, Interpersonal Relationships. The dimensions were initially associated with each question according to the focus of the study. However, during the interviews, each participant was able to approach the questions from their own perspective, sometimes emphasising one dimension over another. Needs and challenges were addressed transversally across the interview guide.

### 2.4 Ethical approval

The study was approved by the Ethics Committee of the Pontifical University of Salamanca (UPSA), as documented in Record No. 14/06/2024. It was conducted in accordance with the principles of the Declaration of Helsinki (63). Ethical standards were upheld throughout the study by ensuring participant anonymity and confidentiality of the data gathered during the interviews. Participants were provided with an explanatory dossier and an informed consent form.

### 2.5 Data analysis

The interview transcripts were analysed using Nvivo 15 software for data storage, organisation, coding, and analysis. The analysis was grounded in an interpretational qualitative analysis (64, 65), combining both deductive and inductive analyses (66, 67). Deductive analysis was used to first organize the data into the purpose of study. Inductive analysis was used to establish the following categories that emerged from reading the information provided by the participants. The specific information categories within the main focus of the study were not defined in advance but emerged only after a thorough analysis of the collected data (68). Within the broader deductive framework of the social factor domains, we conducted an inductive thematic analysis to identify cross-cutting patterns in how participants talked about facilitators and barriers. Following Braun and Clarke (69) approach to thematic analysis, themes were actively generated by the researchers through iterative coding and comparison, rather than assumed to ‘emerge’ automatically from the data. Through this process, ‘needs’ and ‘challenges’ were constructed as analytic categories that captured recurring concerns related to participation in MA basketball.

The data were organised into a hierarchical structure (70), and the constant comparative method (71) was applied to structure the information in a way that facilitated understanding the relationships among the emergent themes. The interviews were coded first into several thematic categories related to the social factor within the QoL paradigm. These included RI, SI, and IR. The coding process followed the principles of thematic analysis (72, 69) and was conducted collaboratively by two researchers, who initially coded the transcripts independently. Their coding systems were then compared and discussed to ensure consistency. Discrepancies were reviewed and resolved in consultation with a third researcher, who served as an external reviewer to reinforce analytic rigour (73, 74). The same process was followed during the interpretation phase, where emerging themes were analysed and refined through a reflective approach.

Data analysis focused on exploring how each of these dimensions influenced participants’ perceived social factor and the identification of the current perceived needs related to their participation in this context. The following qualitative techniques were employed: (1) Data were imported into Nvivo 15, and cases and attributes were created to characterise each participant and allow for individual testimony consultation; (2) An exploratory analysis was performed on the 100 most frequent words (with a minimum of four letters) from the compiled transcript; (3) These keywords were subjected to textual searches to identify the context in which they were used; (4) The analysis followed thematic analysis stages to identify, analyse, and report themes emerging from the interviews (69, 72, 75). The categorisation technique involved transforming data into representative content. The stages were as follows (48): (a) Full reading of the transcripts, (b) Initial categorisation of Meaning Units based on the interview questions, named according to the study terminology, researcher aims, and expertise, (c) Thematic and Categorical reading, reviewing all categorised material, revising coding as needed, and identifying new emerging categories.

In addition, a coding matrix was also taken into account to support data interpretation (Table 3). It presents the number of references made by each participant (P1–P11) across thematic categories and subcategories, facilitating the identification of dominant themes and the distribution of contributions among players, and providing insight into the relative weight and salience of each category within participants’ discourse (76).

**Table 3:** Coding matrix: themes, categories and subcategories emerging from individual interviews.

Categories/subcategories	P1	P2	P3	P4	P5	P6	P7	P8	P9	P10	P11
**Interpersonal relationships**	4	6	9	8	22	13	11	10	15	3	14
Relationship with teammates	2	0	3	2	7	5	3	6	4	2	7
Relationship with coaches	0	0	0	0	3	1	0	1	3	0	3
Relationship with the team outside basketball	2	6	6	6	12	7	8	3	8	1	4
**Social inclusion and rights**	16	19	16	21	22	39	21	9	9	4	10
Community participation	0	4	4	0	1	3	1	0	0	0	2
Social support	5	3	1	6	10	12	8	3	0	3	2
Recognition and social commitment	0	5	3	5	1	6	1	2	1	0	2
Differences between contexts	6	3	4	4	5	3	1	2	3	0	1
Mixed Ability	1	0	0	1	1	5	6	1	1	0	1
Rule compliance	3	4	4	3	3	9	3	1	3	1	2
Rights	1	0	0	2	1	1	1	0	1	0	0
**Barriers, future and proposed changes**	7	15	16	21	6	12	11	0	11	18	5
Competitions	3	3	2	5	3	4	4	0	2	1	2
Time constraints	3	2	2	5	3	2	6	0	1	2	3
Refreshing the team	0	0	2	1	0	6	0	0	0	0	0
Communication and lack of awareness	1	10	10	10	0	0	1	0	8	15	0
**Total**	27	40	41	50	50	64	43	19	35	25	29

Own elaboration.

## 3 Results

**Figure 1:**
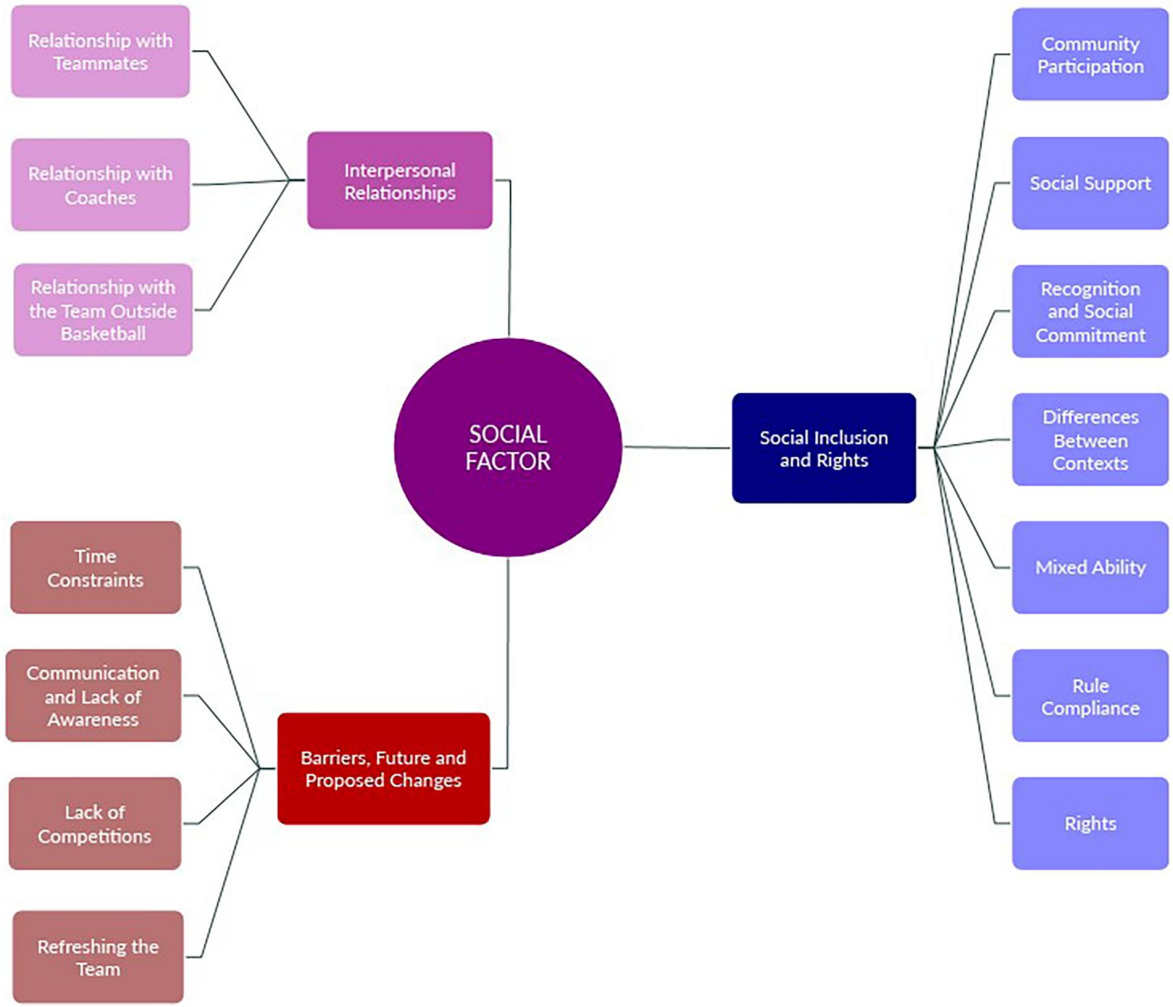
Mind map based on categories and subcategories. Own elaboration.

The conceptual map (Figure 1) presents a visualisation of the data obtained through thematic analysis. From the concept of social factor, themes emerged across the different dimensions. However, RI was merged with SI to facilitate understanding, due to the relatively low number of references under RI. Notably, SI and RI stood out with the highest number of subcategories, totalling seven.

Participants’ interventions revealed significant nuances within each analysed category. The following sections present the thematic findings derived from the analysis, organised according to the established coding framework and supported by illustrative quotations. The results are structured around the main thematic categories and subcategories, highlighting dominant patterns, shared meanings, and points of divergence within participants’ discourse.

### 3.1 Social factor

#### 3.1.1 Interpersonal relationships

Participants’ testimonies revealed that sports practice not only facilitates interactions during training and matches but also fosters meaningful bonds that extend beyond the playing space. Three key areas emerged in this category: peer relationships, relationships with coaches, and relationships outside the basketball context. These dimensions provide insight into how social connections are formed and maintained, the elements that strengthen a sense of belonging and mutual support, and how the sporting environment can act as a catalyst for the development of meaningful interpersonal relationships.

Participants described the basketball team as a positive and inclusive environment that fostered social interaction, respect, and camaraderie (P1, P6–P8). The inclusive atmosphere was highly valued, with one participant explaining: “*I feel good, like just another member of the team. There's a lot of camaraderie, a lot of joy; when you arrive at the locker room, they give you hugs. Both people with and without disabilities, it's a very positive environment”* (P3). Strong bonds of friendship were reported within the group (P3, P5, P9–P11), alongside close relationships with coaches (P8, P9). One player noted: “*The coach is like a second mum. A friend, a second mum […], she's everything”* (P5), while another highlighted the motivating and supportive nature of the team: “*The best thing is that you meet people, play basketball, get a bit more motivated, and teammates always give you a hand”* (P6). Participants also emphasized the value of team cohesion and unity, with one stating: “*I like being with the whole team to chat, to build the team, build unity”* (P7). A female player in particular expressed a strong sense of belonging and a preference for gender specific teams, she explained: “*They treat me well; I like being with the girls’ team”* (P8), reflecting on previous mixed gender experiences: “*The best thing has been the change […]; I want to be with the girls”* (P8).

Interpersonal connections extended beyond the court for all participants. Social activities outside training, such as team dinners (P1–P3, P5–P7, P10, P11) and attending Saski Baskonia first team games, further strengthened group cohesion and a sense of belonging (P4, P7–P9). One participant shared photographs during the interview of teammates and coaches to whom he felt close (P9). The sense of family and belonging was frequently highlighted: “*The family we have formed together. It is very beautiful”* (P11), and “*I love having this team; they encourage me a lot”* (P5).

#### 3.1.2 Social inclusion and rights

Beyond interpersonal ties, participants also described how Mixed Ability basketball enabled them to experience social inclusion and exercise their rights in the sporting context, consistent with the Social Inclusion and Rights domains of the QoL model. Within the MA model, sport was not only perceived as a space for interaction and enjoyment, but also as a setting where fundamental values of equity, respect, and participation were put into practice. Participants’ narratives highlighted several aspects that reflect how they experienced and perceived these processes: community participation, received social support, recognition and commitment from the environment, perceived differences from other sporting contexts, rule compliance, and the experience of exercising their rights.

Access to sport was recognised as a right, with participants emphasising its value beyond participation, as it entails being an active part of society and sharing experiences under equal conditions (P4, P6). In this regard, it was stated that it should “*be a right and involve participation in society, that is what matters most”* (P6). The importance of ensuring that people with disabilities can play alongside teammates without disabilities in line with legal frameworks was underscored (P4). Some players recalled feeling segregated in the past (P6, P9) or having experienced bullying in previous sports settings (P1). However, their involvement in MA enabled them to actively experience inclusion in sport (P1, P6, P9). Phrases such as “*an amazing team feeling”* (P4) and “*now I participate in society”* (P6) were used. A strong sense of community and belonging within the team was valued (P1, P3, P5, P6, P9, P11). Players felt valued without distinction between those with and without disabilities, contributing to an atmosphere of friendship and mutual respect (P3, P4, P7, P9). One player expressed it by saying: “*I'm not a volunteer, I'm an equal player, just like someone with Down syndrome […], I'm just a player like anyone else.”* (P3). Similarly, a rejection of a charitable framing was voiced: “*Socially, yes, it's great to have people with disabilities, but I didn't think of it like* ‘*I'm doing social work.’ No, we’re playing basketball.”* (P2).

Players also emphasised that understanding and applying official rules was essential to feeling fully included in the team (P1, P5, P6, P9). They perceived that playing under the same rules as conventional teams provided an authentic experience, without concessions that might distort the game (P1, P2, P4, P5, P7, P11). In contrast, adapted rules were seen as potentially highlighting differences between players and limiting development (P2–P5, P11). MA was perceived as enabling genuine competition and increasing player engagement in team dynamics (P2–P4, P6, P8).

A dynamic of collaboration was perceived, where all players encouraged and supported each other, reinforcing group cohesion (P1, P2, P6, P7, P11). Several players with disabilities noted that the environment also enabled them to support others—by motivating the team, helping new players, or contributing tactically (P1, P5–P8). Players without disabilities acknowledged and appreciated this support, highlighting the involvement of teammates with disabilities in group dynamics (P2–P4). One player expressed his encouragement to the team by saying: “*Come on, we’re a team, let's go, why be afraid? Let's get back and defend!”* (P6). Public recognition during matches was also appreciated (P5, P6, P9, P11). Some professional Baskonia players attended a game and cheered on the team, which was enthusiastically and proudly received (P2, P3).

### 3.2 Perceived needs and future challenges

This thematic block encompasses the needs and challenges that emerged within the social factor, as well as participants’ suggestions and aspirations for future improvement. These issues cut across the three social factor domains, affecting opportunities to sustain interpersonal relations, to deepen social inclusion and to fully realise rights to participation. The main challenges reported included limited time, the need to rejuvenate the team and communication difficulties and lack of awareness within the team. These elements offered a critical and constructive perspective, contributing to the identification of areas where the programme could be enhanced.

#### 3.2.1 Interpersonal relations and participation opportunities (IR, SI)

Participants stated that one hour of weekly training was insufficient. Therefore, they proposed increasing the duration and frequency of sessions (P1, P2, P4, P6, P7, P9, P10, P11). Regarding this, one player noted that compared to other teams they compete against, “*I would like to train two days, more than just Wednesday […]. Because we just play for one hour and we need to play more”* (P7). Beyond physical performance, players linked training time and competition opportunities to maintaining relationships and feeling part of the group, and many also expressed a desire for more social activities, believing they would further reinforce team bonds (P1, P2, P5, P7, P9, P11). However, several noted that time constraints would make participation difficult (P4, P6): “*day-to-day obligations are what they are; it's not so easy to fit everything in, but, if possible, yes”* (P4). They also suggested introducing new, younger players to the team in order to ensure continuity and maintain competitiveness if a league were to be established (P3, P4, P6). However, some questioned the suitability of forming a league for players attending under parental pressure (P2) or if opponents do not share the MA philosophy (P3, P4). Most players agreed that a league would be a valuable opportunity to improve and give greater continuity to the project (P1, P2, P5, P7, P9, P11). One female player expressed her enthusiasm by stating: “*A competitor. Ambitious to achieve whatever we want”* (P11). Another suggested: “*Mixed Ability matches with teams from Biscay, Gipuzkoa, Navarre”* (P6).

#### 3.2.2 Knowledge, attitudes and inclusive dynamics (SI, RI)

Participants without disabilities reported a general lack of knowledge about the characteristics and specific needs of people with disabilities, which led to insecurity in interactions (P2, P3, P4). This knowledge gap caused uncertainty about certain behaviours, such as discomfort with physical contact, and hindered the development of inclusive dynamics suitable for people with autism or Down syndrome (P2, P3, P4). One player criticised the lack of specific training: “*No one ever told me how to interact with someone with a disability […], they just tell us their name and that's it”* (P2). Although the player's remark could reflect a lack of prior knowledge about interacting with people with disabilities, it may also indicate that the programme does not focus on disability in a generalized or categorical sense. Instead, it appears to adopt a more personalized approach, emphasising getting to know the individual, prioritising personal connection over understanding disability from a global or theoretical perspective. Nevertheless, it is important to note that participants reported not having received any formal training or knowledge regarding the MA approach.

#### 3.2.3 Communication and lack of awareness (IR, SI)

It was observed in the language used during the interviews that participants without disabilities tended to differentiate between the two groups, referring to themselves as “*supporters”* while referring to participants with disabilities as “*them”* (P2–P4). For example, one participant stated, “*both the supporters and the people with disabilities”* (P4). However, this linguistic distinction was not identified by participants with disabilities. Other communication issues were also reported with players who had hearing impairments or lacked fluency in the language, which affected group interaction (P2–P4, P10). One player commented: “*If I don't understand them, then why talk to them?”* (P10). Suggested improvements included incorporating individuals with sign language skills or using visual methods to facilitate communication (P9, P2).

## 4 Discussion

This qualitative study provides an in-depth and contextualised understanding of the perceived changes in the social factor of players from three MA basketball teams, as well as needs and challenges that still present. Regarding the aim of the study, participants expressed diverse perspectives that reflect the multidimensional nature of the topic. Their contributions highlighted key ideas related to the MA model, the factors influencing their perceptions, and the implications of these on their lived experiences. Although all three domains of the social factor were considered in the research question and the analytic framework, participants’ accounts primarily highlighted interpersonal relations and concrete experiences of social inclusion in the team and community. In contrast, explicit references to Rights were less frequent and tended to appear embedded in these relational and inclusion narratives (e.g., “being just another player” or “now I participate in society”), rather than as formal or legal claims about entitlements.

The findings demonstrated that participation in MA basketball has positively impacted participants’ dimensions of QoL linked to the social factor. SI and RI, understood not merely as access but as belonging and active participation, emerged as the most prominent dimensions. As noted by Kiuppis (32) notes, achieving genuine inclusion in mainstream environments requires a participatory and authentic atmosphere. In this sense, social inclusion entails both social connectedness and a genuine sense of belonging (38). Players in this study reported feeling valued and recognised as equals, rejecting paternalistic or charity-based approaches. They described an inclusive coexistence in which no practical differences were perceived between people with and without disabilities. Unlike prior research, this study showed that MA, in the context of Baskonia, successfully fostered genuine SI within a mainstream environment (77, 78). As Simplican et al. (13) suggest, the key components of SI are the interaction between IR and community participation. In this regard, Shapiro and Martin (79) found meaningful associations between social acceptance, close friendships, and overall self-esteem. Similarly, Buchanan et al. (80) highlighted that ongoing physical activity participation largely depended on individual factors, the quality of IR, and community support.

The study's findings reflect three theoretical catalysts proposed by Gómez et al. (6), Verdugo et al. (81), and Verdugo et al. (82): connections, interactions, and facilitating conditions. Connections were evidenced in the meaningful bonds developed by participants with teammates and coaches, strengthened both within and beyond the sports context. The interactions stemming from these connections created mutual natural support systems that promoted inclusion, a sense of belonging, active participation, and personal development. In this regard, Navas et al. (83), found that the benefits of deinstitutionalisation were not solely due to a change in setting, but rather the sustained presence of supports that encouraged decision-making. Similarly, this study highlighted the context as an environment in which people with disabilities could also provide support autonomously. This aligns with the shared citizenship paradigm, which asserts that people with disabilities should be fully included, recognised, and valued as active, equal, and contributing members in all areas of social life (84, 85). Finally, facilitating conditions, such as programme structure, environmental attitudes and recognition, and an inclusive approach, created an accessible and affirming space that enhanced both functionality and well-being for all players. The IR described by participants revealed a strong sense of community, group cohesion, and genuine friendship, extending beyond sports participation. In this vein, Blick et al. (86) found that people with disabilities engaged in physical activity tended to go out into the community more often, which was associated with greater participation and SI compared to those who were inactive. Previous studies on MA support this, showing that the model encourages social connections, interactions, and the development of networks and friendships (39, 42–44, 46). However, Jeanes et al. (35), warned that interactions between athletes with disabilities and other club members tend to be limited, and that inclusive activities are often organised separately from mainstream club operations. In contrast, the MA team analysed in this study was part of an established basketball network, which facilitated their inclusion and active participation in a shared sports environment.

Consistent with the findings of Wilson et al. (87) and Fraser et al. (88), participants reported a transition from feeling excluded to actively participating, supported by the provided support system. Thus, participation in the MA enabled an experience of active inclusion in spaces previously perceived as inaccessible, aligned with the effective exercise of their right to participation. These results also support the assertions of Misener and Darcy (30), who highlight the need for a range of community options so individuals can freely decide how and in what context to engage in activity. Several participants stated that playing under adapted rules would be unsatisfying, as they believed it could limit their learning and undermine the authenticity of the experience. Pearce and Sanderson (36), argued that adapting rules without considering personal preferences may result in superficial inclusion, where people with disabilities do not experience full social or personal development. In this study, participants especially emphasised the importance of knowing and understanding official rules, which allowed them to enjoy their rights and participate on equal terms, feeling part of the game. Similarly, other studies on MA highlights the need to participate without modifications to the official rules (39, 42). Therefore, to achieve inclusive and accessible experiences in mainstream settings, it is crucial to introduce reasonable adjustments tailored to individual needs (36, 89). In this sense, it is essential to address disability characteristics and natural support needs on a case-by-case basis to guarantee meaningful participation (6, 90).

Among the main challenges identified, participants highlighted the need to increase training frequency and competition opportunities, as limited time could restrict their social network contact. Likewise, Elipe-Lorenzo et al. (42) noted that most participants expressed a strong desire to see the team again once they returned home; however, the majority indicated that they only saw their teammates once or twice a week. Similarly, Nettleton et al. (91) reported that overall activity levels and frequency were low, with participants expressing a desire for more sports involvement. From this perspective, time constraints in training schedules hinder the strengthening of friendships within the sporting context (79). As Asselt-Goverts et al. (92) argue, frequency of contact is a fundamental aspect in the structure of IR. Furthermore, this study also revealed difficulties in communication with some teammates, which impacted group cohesion and limited IR. Moreover, the “us vs. them” mentality often leads to exclusion in sport—driven by ignorance or segregative social dynamics (42, 43, 46, 93). As in the study of Dyer and Sandford (46) this form of division was primarily observed among participants without disabilities, rather than among people with disabilities. Additionally, several participants reported having limited or no prior knowledge about inclusive practices before to their involvement. This finding suggests that specific training in MA may be necessary, particularly given that the team did not receive any formal preparation. Other studies have noted that communicative differences can affect social synchrony and the sense of belonging within a group (94, 95). In this regard, for SI to be effective, it must overlap with and reinforce IR (13). Similarly, Corazza and Dyer (39) highlight through MA participants’ experiences that social inclusion is a dynamic process, influenced both by the individual–context relationship and the interplay between interpersonal relationships and community participation. Evidence from other MA studies indicates that the model was perceived as a valuable tool for fostering inclusive learning and participation within sporting contexts (45, 96, 97). Future research could explore whether providing formal training in the MA approach influences participants’ behaviours and attitudes, and whether this might contribute to more effective inclusive practices.

## 5 Limitations

Despite the richness of the findings, this study presents several limitations that should be considered when interpreting the results. Firstly, the close relationship established between the researchers and the players, while it facilitated trust and openness during the interviews, may have introduced a social desirability bias, as participants might have responded more positively or avoided direct criticism of the team or coaches. Although efforts were made to maintain a neutral stance, this type of influence is difficult to eliminate entirely in qualitative research contexts. Moreover, theoretical saturation was not fully achieved in the rights dimension, as no additional participants were available for interview. Therefore, findings related to this aspect should be interpreted with caution.

Secondly, the qualitative design employed does not allow for statistical generalisation to other populations or settings. Additionally, the cross-sectional approach adopted does not permit an accurate assessment of the temporal evolution of the described effects. The analysis focuses on specific perceptions gathered at a particular moment within the sporting participation process. Longitudinal research would be valuable to explore how these changes consolidate, transform, or diminish over time, as well as their sustained impact on participants’ QoL and life trajectories.

## 6 Conclusion

Overall, the findings of this study demonstrate that MA basketball can be far more than a sporting activity—it may be regarded as a powerful tool for enhancing interpersonal relationships, promoting social inclusion, and empowering the rights of people with disabilities. However, achieving full inclusion requires overcoming persistent challenges such as limited communication between some teammates and the low frequency of training sessions and competitions. This highlights the need for individualised reasonable adjustments and sustained initiatives that not only ensure physical access but also promote active, valued, and self-determined participation. Ultimately, inclusion should not be understood as a static goal, but as a dynamic and ongoing process, shaped both by environmental structures and the quality of human relationships within them. Future studies should adopt longitudinal approaches to examine how the social impacts of the MA model evolve over time and explore the replicability of this study in other contexts and sporting disciplines.

## Data Availability

The raw data supporting the conclusions of this article will be made available by the authors, without undue reservation.
